# Chronic heat stress promotes liver inflammation in broilers via enhancing NF-κB and NLRP3 signaling pathway

**DOI:** 10.1186/s12917-022-03388-0

**Published:** 2022-07-23

**Authors:** Yi-Lei Liu, Kang-Ning Ding, Xing-Ling Shen, Han-Xiao Liu, Yi-An Zhang, Yu-Qing Liu, Yong-Ming He, Lu-Ping Tang

**Affiliations:** School of Life Science and Engineering, Foshan University, Foshan, 528225 China

**Keywords:** Heat stress, Broilers, Liver, NF-κB, NLRP3

## Abstract

**Background:**

This study investigated the effects of chronic heat stress on liver inflammatory injury and its potential mechanisms in broilers. Chickens were randomly assigned to the 1-week control group (Control 1), 1-week heat stress group (HS1), 2-week control group (Control 2), and a 2-week heat stress group (HS2) with 15 replicates per group. Broilers in the heat stress groups were exposed to heat stress (35 ± 2 °C) for 8 h/d for 7 or 14 consecutive days, and the rest of 26 hours/day were kept at 23 ± 2 °C like control group broilers. Growth performance and liver inflammatory injury were examined for the analysis of liver injury.

**Results:**

The results showed that heat stress for 2 weeks decreased the growth performance, reduced the liver weight (*P <* 0.05) and liver index (*P <* 0.05), induced obvious bleeding and necrosis points. Liver histological changes found that the heat stress induced the liver infiltration of neutrophils and lymphocytes in broilers. Serum levels of AST and SOD were enhanced in HS1 (*P <* 0.01, *P <* 0.05) and HS2 (*P <* 0.01, *P <* 0.05) group, compared with control 1 and 2 group broilers. The MDA content in HS1 group was higher than that of in control 1 group broilers (*P <* 0.05). Both the gene and protein expression levels of HSP70, TLR4 and NF-κB in the liver were significantly enhanced by heat stress. Furthermore, heat stress obviously enhanced the expression of IL-6, TNF-α, NF-κB P65, IκB and their phosphorylated proteins in the livers of broilers. In addition, heat stress promoted the activation of NLRP3 with increased NLRP3, caspase-1 and IL-1β levels.

**Conclusions:**

These results suggested that heat stress can cause liver inflammation via activation of the TLR4-NF-κB and NLRP3 signaling pathways in broilers. With the extension of heat stress time, the effect of heat stress on the increase of NF-κB and NLRP3 signaling pathways tended to slow down.

**Supplementary Information:**

The online version contains supplementary material available at 10.1186/s12917-022-03388-0.

## Background

The poultry industry is growing across the world to fulfill the increasing demands of poultry meat and eggs. Heat stress is a top environmental concern in poultry industry worldwide, being potentially triggered by a variety of conditions, such as failure of ventilation, climatic conditions, stocking density, humidity and temperature controls. Among them, high ambient temperature plays a significant role Heat stress jeopardizes human and animal health and results in major economic losses in public health care and livestock production [1]. When the body exposed with heat stress, body temperature, respiratory rate, heart rate and rectal temperature will increase adaptively, which will affect the feed intake and production efficiency of poultry, thus adversely affecting the production economy [2]. In addition, long-term genetic selection of poultry for improved performance induced higher metabolic heat production and increased sensitivity to heat stress [3]. Research has shown that heat stress causes average annual economic losses of 128–240 million dollars for poultry industries in the United States, and the unfavorable influence has progressively increased as global temperatures rise [4]. Alleviating heat stress is important to reduce the economic loss of the livestock industry.

The liver is sensitive to ambient stress [5]. Research has found that heat stress can induce liver oxidative stress and reduce the immune responses of laying hens, which results in decreased poultry production performance, such as reduced body weight and food consumption [6]. Continuous exposure to high temperature in broilers can lead to liver tissue damage and decrease nonspecific immunity [7] and have other negative effects on the immune response [8]. Moreover, the intestinal stress caused by high temperature results in bacterial translocation and unbalanced intestinal flora and induces intestinal endotoxin entry into the liver through the internal circulation [9].

Innate immune cells in the liver recognize dangerous substances or damaged cells through pattern recognition receptors (PRRs), thus activating TLR4 signalling to promote inflammatory responses [10]. NF-κB, which acts downstream of TLR4 [11] and other immune receptors [12], can increase the overproduction of proinflammatory interleukin 6 (IL-6), IL-1β and tumour necrosis factor-alpha (TNF-α), which leads to the occurrence of inflammatory response [13]. NF-κB activates NLRP3 while inducing a variety of inflammatory chemokines [14], cytokines and cytokine precursors, including pre-IL-1β, and is therefore important for inflammasome initiation and assembly [15]. However, whether heat stress mediates inflammation in the liver of broilers and its relationship with NF-κB-NLRP3 are still unclear. In this study, we designed a model of chronic heat stress in broilers to investigate whether heat stress activates NF-κB-NLRP3 to exacerbate liver inflammation.

## Results

### Chronic heat stress inhibited the growth performance of broilers

The growth performance indices of broilers mainly include body weight growth and feed conversion capacity. The daily body weight and feed intake of broilers were monitored in this study, and the results were shown in Table 1. Compared with the control 2 group, the body weight gain of the broilers in the HS2 group was significantly decreased (from 412.95 ± 96.01 to 300.01 ± 36.50, *P <* 0.01), the feed intake and feed conversion ratio were also inhibited by heat stress, but the effect was not significant. The above 3 indicators had no significance between control 1 group and HS1 group. With increased heat stress time, these adverse reactions gradually enhanced, the growth performance of the broilers was significantly affected by heat stress for 2 weeks.

**Table 1:** Effects of heat stress on growth performance of broilers

Growth Performance					
Treatment	Initial Body Weight	Final Body Weight	ADG	ADFI (g)	FCR
Control 1	352.52 ± 18.42	539.64 ± 23.69	187.11 ± 13.25	401.13 ± 5.30	2.15
HS1	353.26 ± 16.08	496.14 ± 28.83^**^	143.77 ± 18.76	255.99 ± 16.38	2.52
Control 2	354.78 ± 24.23	773.67 ± 97.37	412.95 ± 96.01	847.89 ± 15.62	2.22
HS2	355.49 ± 15.43	655.50 ± 40.06^**##**^	300.01 ± 36.50^**##**^	758.74 ± 11.95	2.55

^**^P < 0.01 vs. control 1, ^**##**^*P <* 0.01 vs. control 2

### Effects of heat stress on liver necropsy and histological changes in broilers

**Fig. 1:**
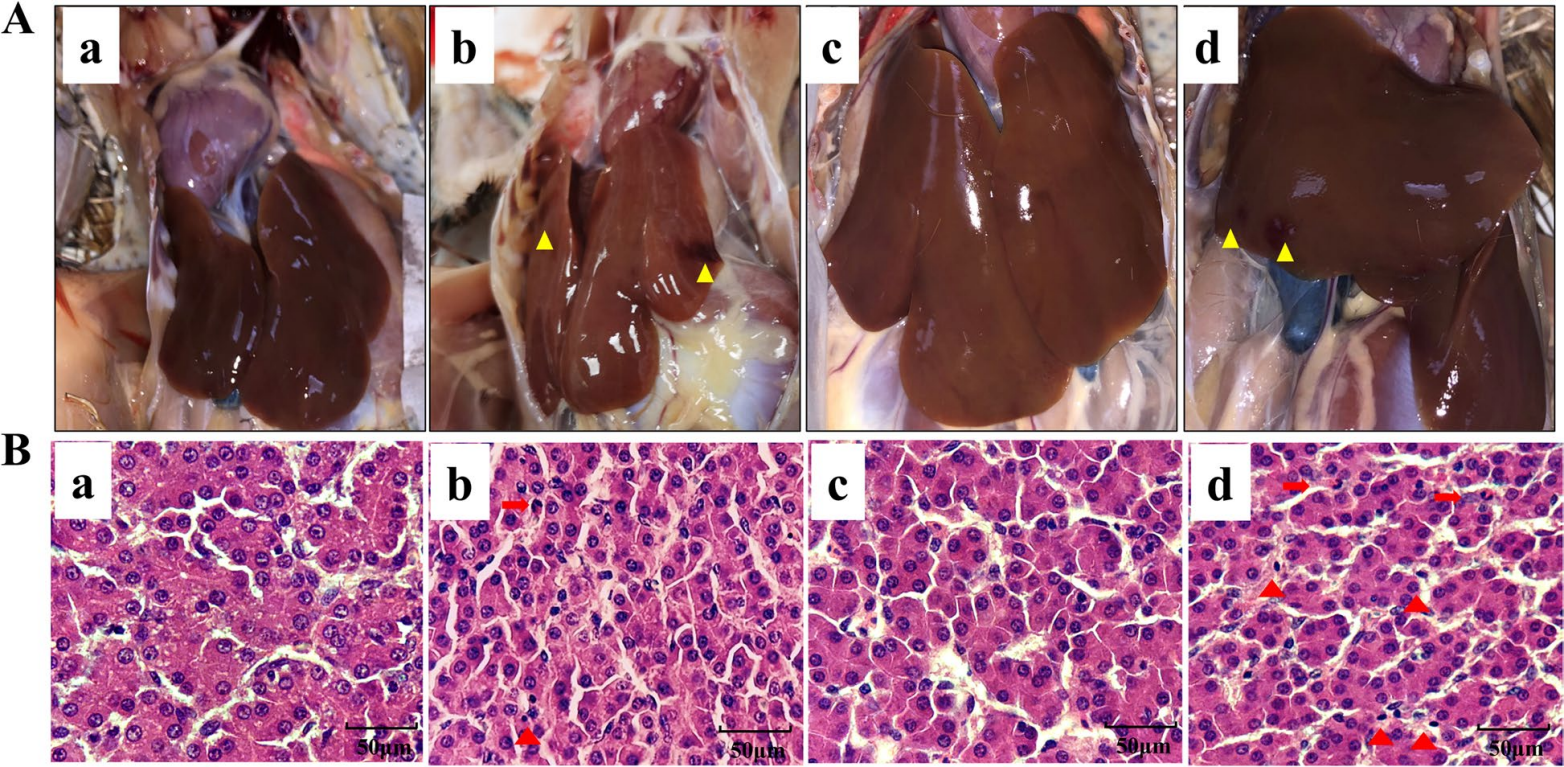
Effects of heat stress on liver changes of broilers. **A** Liver necropsy changes. (a) control 1 group; (b) HS1 group; (c) control 2 group; (d) HS2 group Yellow triangle represents necrosis points. **B** Liver histological changes. Red arrow represents neutrophils infiltration. Red triangle represents lymphocytes infiltration

As shown in Fig. 1, the livers of the broilers after necropsy were observed that the texture of the liver surface in the broilers of the control 1 group (a) and the control 2 group (c) was uniform, without obvious bleeding and necrosis points. HS1 group (b) and HS2 group (d) broilers showed obvious bleeding and necrosis points on the liver surface. Liver histological changes were observed in Fig. 1B. There were no alterations in the morphology of liver in control group broilers. However, compared with the control group broilers, the infiltration of neutrophils and lymphocytes were observed in heat stress group broilers. The results showed that heat stress had a promotion on liver tissue damage.

### Effects of heat stress on liver index and enzymatic activity changes in broilers

**Fig. 2:**
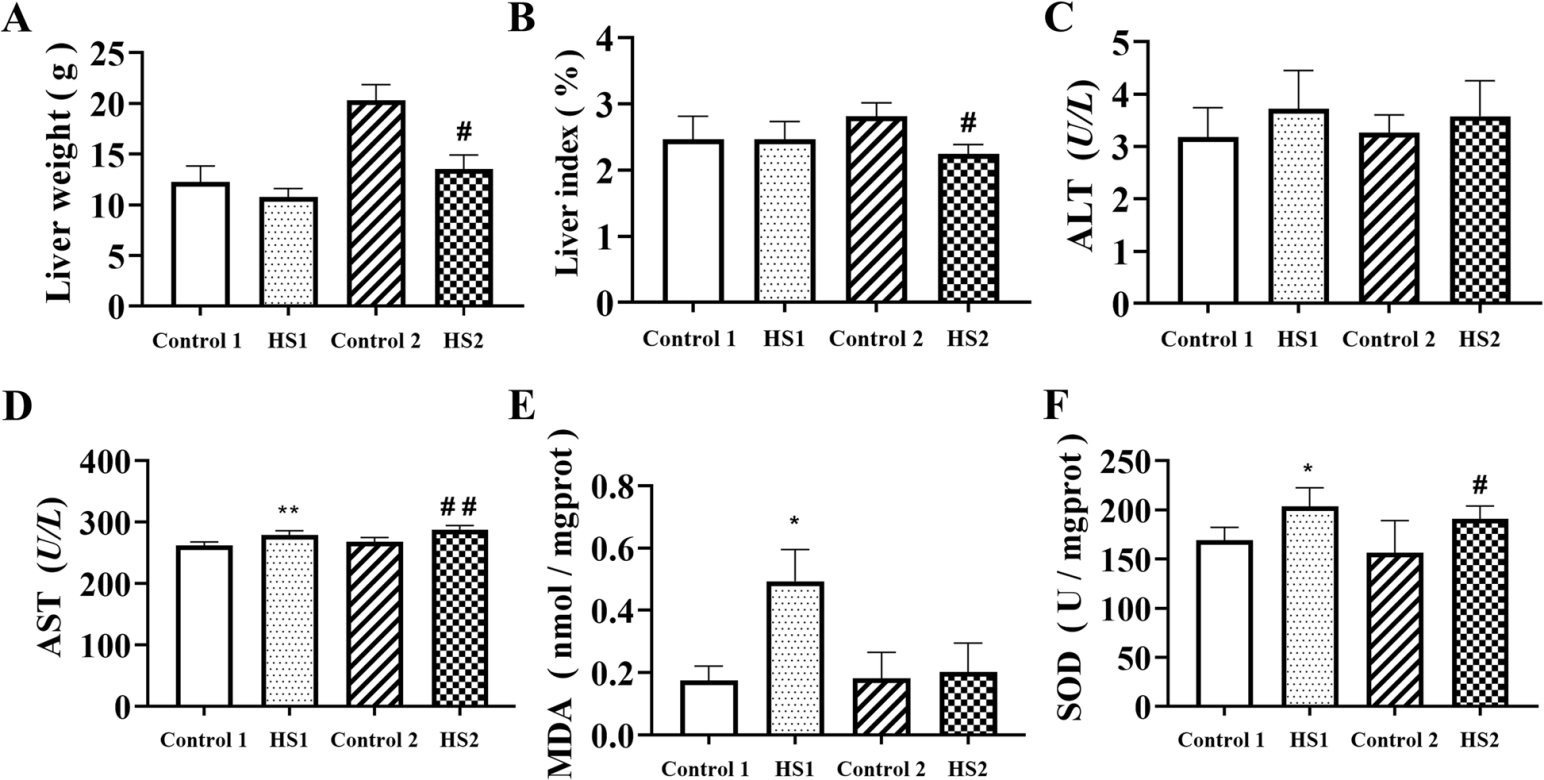
Effects of heat stress on liver of broilers. **A** Liver weight of broilers. **B** Liver index of broilers. Serum (**C**) ALT and (**D**) AST levels. Liver (**E**) MDA and (**F**) SOD level in broilers. ^*^P < 0.05, ^**^P < 0.01 vs. control 1. ^#^*P <* 0.05, ^##^*P <* 0.01 vs. control 2

Liver weight and liver index were measured (Fig. 2) and the results showed that heat stress for 2 weeks significantly reduced the above 2 indicators (*P <* 0.05, *P <* 0.05, respectively), compared with control 2 group. But, the 2 indicator above had no significance between control 1 group and HS1 group (Fig. 2). Compared with control 1 or 2 groups, heat stress for 1 or 2 weeks significantly enhanced serum AST and SOD levels, but had no change in ALT level. The MDA content in HS1 group was higher than that of in control 1 group broilers (*P <* 0.05).

### Chronic heat stress enhanced the protein expression of TNF-α and IL-6 in the livers of the broilers

**Fig. 3:**
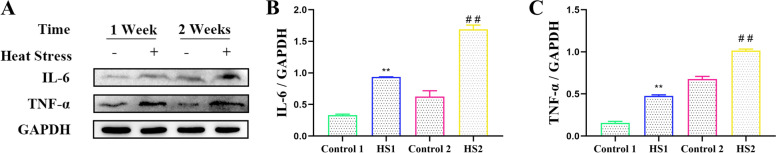
Effects of heat stress on liver proteins levels of TNF-α and IL-6 in broilers. **A** Protein expressions of liver TNF-α and IL-6 in broilers. Relative expressions of (**B**) IL-6 and (C) TNF-α. ^**^P < 0.01 vs. control 1; ^##^*P <* 0.01 vs. control 2

As shown in Fig. 3, heat stress significantly increased the protein expression of IL-6 in the liver after 1 and 2 weeks of heat stress compared with those of the control 1 and 2 groups (*P <* 0.01, *P <* 0.01, respectively). The TNF-α protein levels in the broilers subjected to heat stress for 1 and 2 weeks were higher than that of in control 1 and 2 group broilers (*P <* 0.01, *P <* 0.01, respectively).

### Chronic heat stress activated the NLRP3 inflammasome in livers broilers

**Fig. 4:**
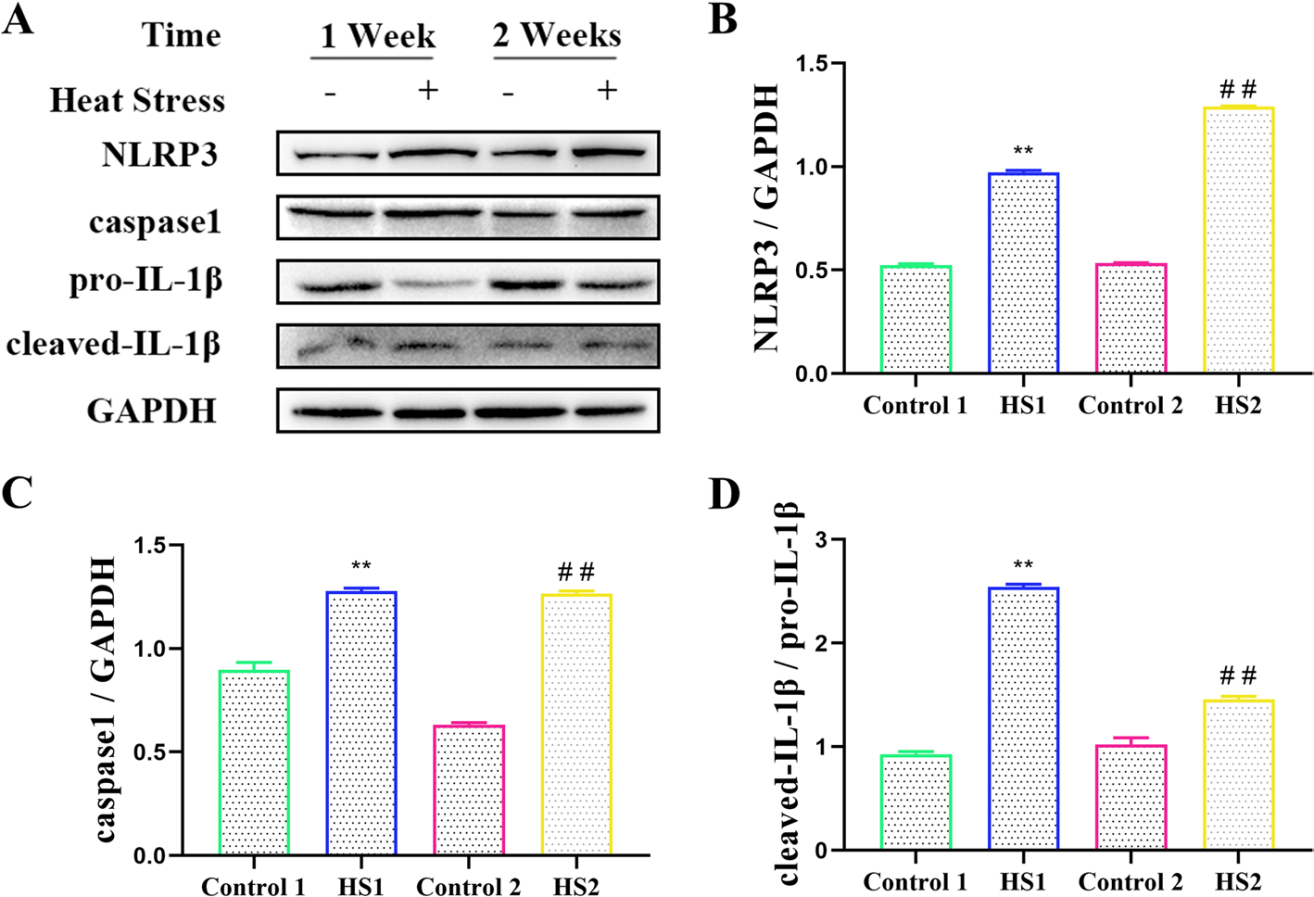
Effects of heat stress on protein expressions of NLRP3 pathways in broilers. **A** Protein expressions of NLRP3 pathways in liver of broilers were measured by western blot. Relative expression of (**B**) NLRP3, **C** caspase 1 and (**D**) cleaved-IL-1β/pro-IL-1β. ^**^P < 0.01 vs. control 1; ^##^*P <* 0.01 vs. control 2

As shown in Fig. 4, the NLRP3 and caspase 1 protein levels in the HS1 and HS2 groups were both significantly increased compared with those of the control 1 and 2 groups (*P <* 0.01, *P <* 0.01, *P <* 0.01, *P <* 0.01). The levels of cleaved IL-1β/pro-IL-1β in the livers of the broilers in the HS1 and HS2 groups were obviously higher than that of in the control 1 and 2 groups (*P <* 0.01, *P <* 0.01). The trend of cleaved IL-1β/pro-IL-1β was consistent with the protein expression of NLRP3 and caspase 1.

### Chronic heat stress strengthened NF-κB pathways in the livers of the broilers

**Fig. 5:**
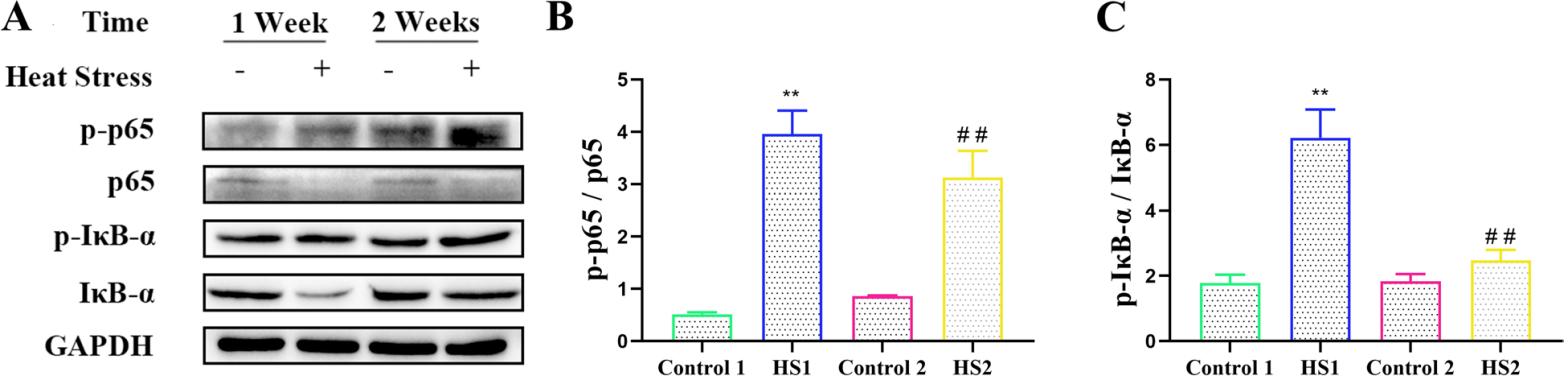
Effects of heat stress on NF-κB pathways in liver of broilers. **A** Protein expressions of NF-κB pathways in liver of broilers. Relative expression of (**B**) p-p65/p65 and (**C**) p-IκB-α/IκB-α. ^**^P < 0.01 vs. control 1; ^##^*P <* 0.01 vs. control 2

We simultaneously detected the expression of NF-κB, IκB-α and their phosphorylated proteins. The results are shown in Fig. 5. Compared with the control 1 and 2 groups, heat stress for 1 and 2 weeks significantly increased the ratio of phosphorylation of NF-κB to NF-κB (*P <* 0.01 and *P <* 0.01, respectively) and enhanced the ratio of phosphorylation to unphosphorylation of IκB-α (*P <* 0.01 and *P <* 0.01, respectively) .

### Chronic heat stress promoted the protein expression of TLR4 and HSP70 in the livers of the broilers

**Fig. 6:**
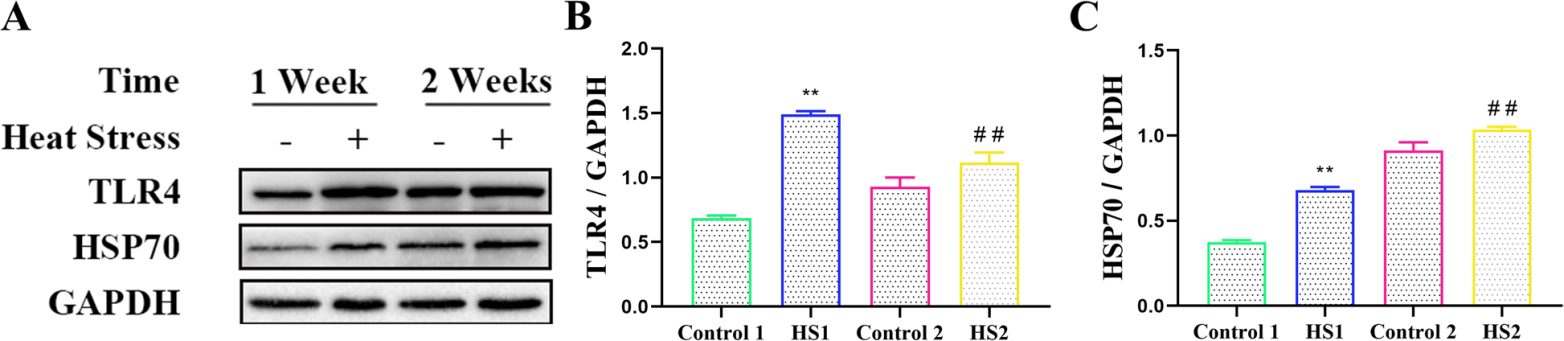
Effects of heat stress on proteins expressions of TLR4 and HSP70 in liver. **A** Proteins levels of TLR4 and HSP70 in liver of broilers. Relative expression of (**B**)TLR4 and (**C**) HSP70. ^**^P < 0.01 vs. control 1; ^##^*P <* 0.01 vs. control 2

To confirm the dependence of inflammation responses on these key factors, the relative gene expressions of TLR4, NF-kB, and Hsp70 in liver of broilers were examined. As shown in Fig. 6, the protein expressions of TLR4 and HSP70 in the liver of the broilers was measured. Heat stress for 1 and 2 weeks significantly upregulated the protein expression of TLR4 compared to that of the control 1 and 2 groups (*P <* 0.01 and *P <* 0.01, respectively). The HSP70 protein has a protective effect on cells, and its nonspecific expression can be increased when the body is subjected to stress. Our results showed that compared with that of the control 1 and 2 groups, the protein expression of HSP70 in the HS1 and HS2 groups were enhanced (*P <* 0.01 and *P <* 0.01, respectively).

### Chronic heat stress upregulated the gene expression of TLR4, HSP70 and NF-κB in the livers of the broilers

**Fig. 7:**
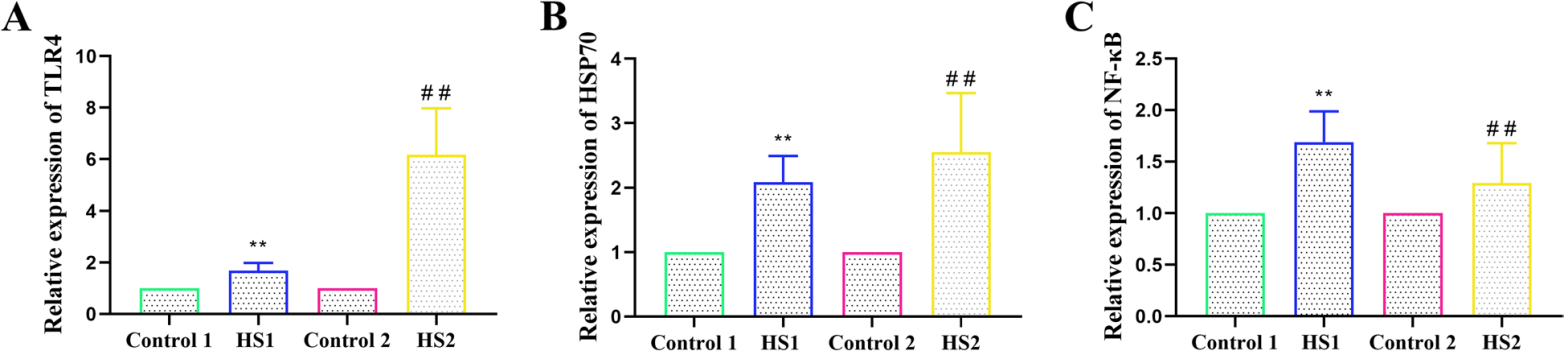
Effects of heat stress on genes expressions of TLR4, HSP70 and NF-κB in liver of broilers. **A** TLR4, **B** HSP70 and **C** NF-κB genes expressions in liver were measured by qRT-PCR. ^**^P < 0.01 vs. control 1; ^##^*P <* 0.01 vs. control 2

As shown in Fig. 7, heat stress for 1 and 2 weeks enhanced TLR4 gene expression (*P <* 0.01 and *P <* 0.01, respectively), especially at 2 weeks of heat stress. An increase in HSP70 mRNA expression was observed in the liver after heat stress for 1 and 2 weeks compared to that of the control 1 and 2 groups (*P <* 0.01 and *P <* 0.01, respectively). Consistently, NF-κB gene expression was also significantly upregulated during heat stress.

## Discussion

High temperatures or hot climates have adverse impacts on the growth performance of poultry due to their higher water intake and lower feed intake. Moreover, high temperature affects the digestive and absorptive function of the gastrointestinal tract and results in a significantly lower weight gain (WG) and feed conversion rate (FCR). High temperature also causes tissue damage, especially damage to the intestine and liver, which affects the normal function of poultry [16, 17]. Heat stress for 2 week decreased weight gain, and heat stress for 1 and 2 weeks reduced the body weight of broilers. The relative organ weight reflects the growth and development of organs to some degree and then affects their functions [18]. Under continuous heat stress, the organ index of the liver and other immune organs and cellular immunity decrease [17, 19], which was consistent with our results. In this study, heat stress for 2 weeks decreased the body weight, liver weight and liver index of the broilers, indicating that heat stress impaired liver growth and development in the broilers. Moreover, the heat-stressed broilers showed obvious bleeding and necrosis points on the liver. Liver histological changes indicated that heat stress induced the infiltration of neutrophils and lymphocytes in liver of heat stress group broilers. As the largest digestive organ in the broilers, liver injury may affect the digestive and absorption functions of the body, which may explain why the growth performance of the broilers decreased to a certain extent after heat stress in the experiment. However, the relationship between the inflammatory cell infiltration of the broiler and susceptibility to heat stress is not fully understood.

One organ capable of exerting strong influence on both bird growth and thermoregulation is the liver. This organ has recently proved effective as a subject for studies for its highly susceptible to heat stress. The overexpression of HSP70 is an indicator of various stress responses, including heat stress. In this study, increased HSP70 mRNA and protein levels were observed in the livers of the broilers in the heat stress group. Extracellular or exosomal-bound HSP70 binds to TLR2 or TLR4 to activate the inflammatory response in animals [20, 21]. TLR4 is thought to be the endotoxin receptor that receives the validation response [22]. Activation of TLR4 stimulates the associated inflammatory signalling pathways. Research has shown that heat stress can significantly upregulate the TLR4 mRNA expression in the liver of broilers [17]. Activation of TLR4 can activate NF-κB, cause the synthesis and secretion of proinflammatory cytokines, and further amplify the inflammatory response. Research has shown that NF-κB protein expression is significantly increased by heat stress [23]. Our results showed that heat stress for 1 and 2 weeks upregulated the gene and protein expression of TLR4 and NF-κB, and heat stress also increased the ratio of NF-κB and IκB-α protein phosphorylation to nonphosphorylation. We further detected the expression levels of inflammation-related proteins in the liver. Our results showed that heat stress upregulated TNF-α and IL-6 protein levels in the livers of the broilers. Therefore, heat stress activates the NF-κB signalling pathway and promotes the secretion of the inflammatory factors TNF-α and IL-6, leading to the occurrence of inflammation in the liver of the broilers.

Heat stress can disrupt metabolic homeostasis in the liver and throughout the system, and the disorder is associated with inflammation [24]. NLRP3 inflammasome is a type of multiprotein complex, and its abnormal activation and regulation are related to the development of various inflammatory diseases. Yang’s study found that the NF-κB/NLRP3 signalling pathway is inhibited in response to acute heat stress [25], and Greene’s study also confirmed that heat stress can reduce the NLRP3 inflammasome [26]. However, most studies have shown that NLRP3 protein levels increase with heat stress temperature [27]. Pei’s study showed that the protein level of NLRP3 increased with the extension of exposure time under heat stress [28]. Studies have shown that stress triggers activation of the NLRP3 inflammasome [29], and excessive accumulation of the inflammasome can trigger inflammation, which mediates liver injury [30]. In addition to NLRP3 activation, heat stress can also promote the activation of the NF-κB signalling, P38, and ERK pathways [31, 32]. Although, there are lots of researches on inflammatory diseases and NLRP3 inflammasome in mice and human, few studies on the changes of NLRP3 inflammasome in liver of broilers treated with heat stress. Therefore, we observed protein changes in the NLRP3 pathway of the inflammasome in heat stressed broilers. The results showed that heat stress significantly increased the NLRP3 protein in the liver of broilers, and the protein levels of the NLRP3 downstream genes caspase-1 was also increased in the heat stress group, compared with the control group. The content of IL-1β precursor was significantly decreased, while the content of IL-1β in the mature and caspase-1 were significantly increased. The results indicated that heat stress activated NLRP3 pathway and the activation of NLRP3 inflammasome eventually leads to the activation of caspase1 and the secretion of IL-1β.

## Conclusion

**Fig. 8:**
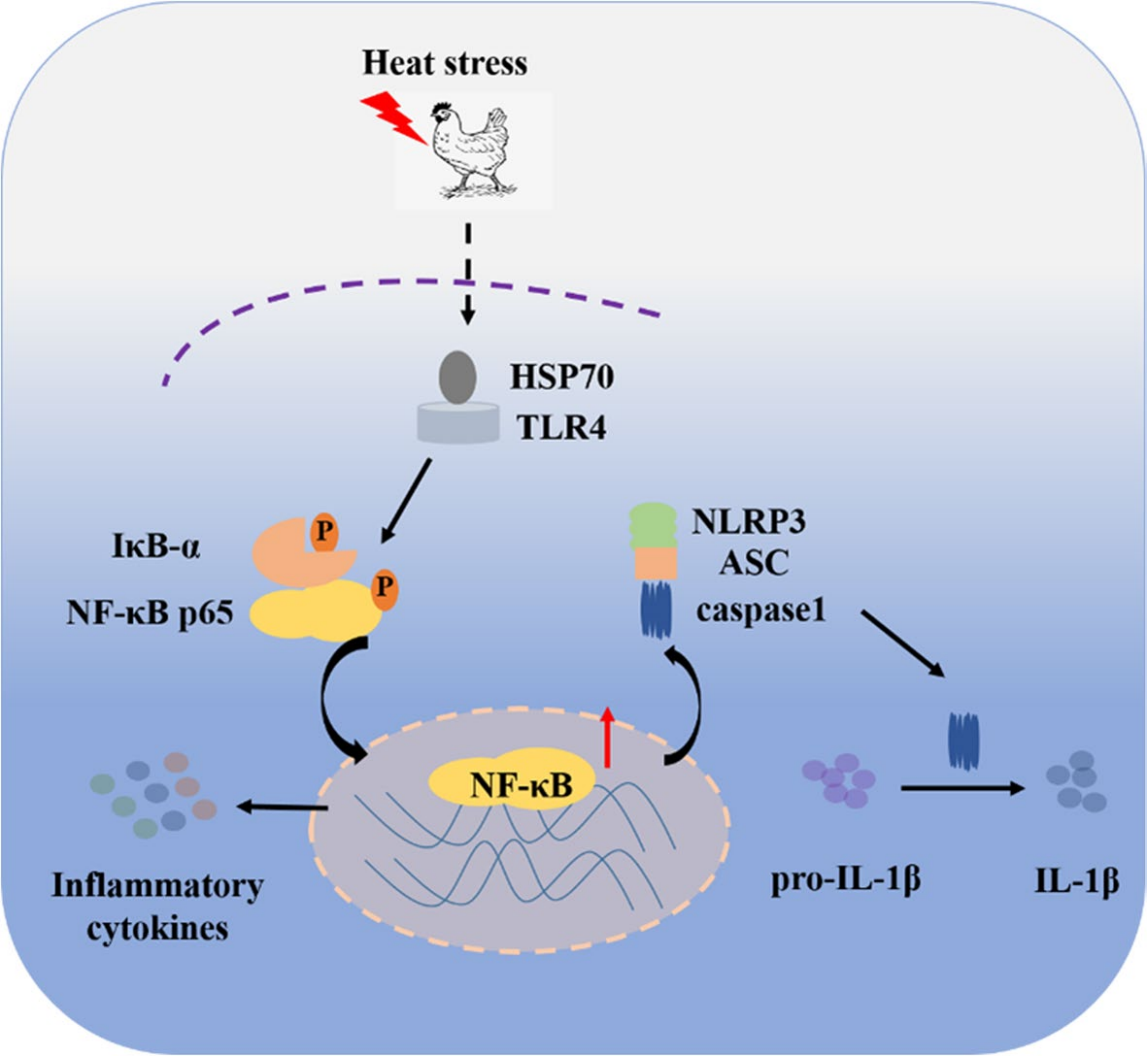
Mechanism of heat stress induced liver inflammation injury of broilers. Heat stress activates TLR4-NF-κB and NLRP3 signaling pathway, which induced the secretion of pro-inflammatory factors

In this study, heat stress inhibited broiler growth performance, increased liver inflammatory cell infiltration, and promoted the release of inflammatory factors in liver of broilers. The expression of TLR4 in the livers of broilers under heat stress was similar to the expression of NLRP3 and the inflammatory factors NF-κB, indicating that heat stress promoted liver inflammatory injury via activation of the TLR4-NF-κB and NLRP3 signaling pathways in broilers (Fig. 8).

## Methods

### Animals treatment

Sixty two-week-old Ma chickens .were purchased from a commercial hatchery (Nanhai Poultry Corporation, Foshan, China). Ma chickens are a pure line of local Qing Yuan Ma Chickens. One week of adaptive feeding, twenty-one-day-old male broilers were randomly divided into 1-week control group (Control 1), 1-week heat stress group (HS1), 2-week control group (Control 2) and 2-week heat stress group (HS2), with 15 replicates in each group. The control group was kept at 23 ± 2 °C, while the heat stress group was kept at the 35 ± 2 °C, and the humidity control as about 70%. The broilers of heat stress groups continuous treatment with high temperature for 8 h every day (8:00–16:00 every day) for 1 or 2 weeks. All broilers had freely drinking water and basal diet. The broilers feed were provided by WENS FOODSTUFF GROUP CO.,LTD, including corn, wheat, soybean meal, soybean oil and other conventional feed additives, amino acids, trace elements and vitamins. We recorded the body weight and the amount of feed consumed every day which were used to analyze the average weight gain (WG), feed intake and feed conversion ratio (GFR). At the end of heat stress, blood samples were taken from the jugular vein and used for the measurement of alanine aminotransferase (ALT) and aspartate aminotransferase (AST), all broilers were sacrificed quickly by CO_2_ inhalation, and liver samples were collected for subsequent testing.

### Pathological changes and the organ index of liver

The liver tissues of broilers were photographed to observe the changes of pathological injury during the slaughter of broilers. Complete liver was taken, washed with autoclaving saline, dried with absorbent paper, weighed and recorded for calculating organ index (organ index = (liver weight / body weight) × 100%).

The liver tissue was fixed with 4% paraformaldehyde for 48 h and dehydrated with ethanol, and then the tissue was embedded in paraffin. The liver wax block was cut into slices (5 μm). Sections were subjected to hematoxylin and eosin (H&E) staining and then observed under an optical microscope (Bio-Rad, USA).

### Enzymatic activity analysis

Serum ALT and AST levels were analyzed using Alanine aminotransferase Assay Kit (C009–2-1) and the Aspartate aminotransferase Assay Kit (C010–2-1). Liver malondialdehyde (MDA) and superoxide dismutase (SOD) levels were measured with malondialdehyde (MDA) assay kit (A003–1-2) and superoxide dismutase (SOD) assay kit (A001–3-2) The four kits were provided by Nanjing Jiancheng Bioengineering Institute (Nanjing, China).

### Western blot analysis

Liver samples were extract with a lysate containing RIPA and phosphatase-inhibitors and phenylmethylsulfonyl fluoride (PMSF) on ice, supernatant protein quantification by BCA kit (Beyoutime, Shanghai, China) before centrifuged at 13000 r/min at 4 °C for 10 min. Proteins (20–40 μg from each sample) were separated on SDS-PAGE gels and transferred to polyvinylidene fluoride (PVDF) membrane. Then, the membrane was blocked with 5% skimmed milk (skim milk powder in Tris-buffered saline-Tween 20) for 1 hour and incubated with primary antibodies (IL-6, TNF-α, NLRP3, caspase 1, pro-IL-1β, cleaved- IL-1β, p-NF-κB p65, NF-κB p65, p-IκB-α, IκB-α, TLR4, HSP70 and GAPDH. Cell Signaling Technology, Danvers, MA, USA). Subsequently, the membrane was incubated with secondary antibodies which contain peroxidase-conjugated, followed by visualized with the Chemiluminescence System. The relative intensity of target protein was analyzed by the Image J software.

### Quantitative real-time PCR

Total RNA from the liver tissue using the Trizol reagent (Ambion, Austin, TX, USA), and reversely transcribed through 1st Strand cDNA Synthesis kit (Takara, Tokyo, Japan) according to the protocol. Then, quantitative real-time polymerase chain reaction using the TB Green™ Premix Ex Taq™ II (Takara, Tokyo, Japan) and a Roche LightCycler 480 system (Roche, Basel, Switzerland). Gene mRNA levels were normalized using the expression of housekeeping gene β-actin and the relative fold changes were calculated using the 2^-ΔΔCt^ method. All primers used for qRT-PCR are listed in Table 2.

**Table 2:** The primers used in qPCR are as Table 2 follows

Gene	GenBank	Primer sequences(5′-3′)	Produce size (bp)
TLR4	NM_001030693	F: GATGCTCTCTATGGGCTTCTCTGATG R: GAGGCTGCTTGGAATACTGGATGG	132
HSP70	AY178442.1	F: CAGGGCAATGCTAGTGTGTACTCATC R: AGGGTCTTTCTTTGGTGTGTTCATACG	120
NF-κB	NM_205134	F: CGAGTGCTTTGTCTACGAGATGGAG R: AGGTCAGCCGCTTCAATCTTCTTC	131
β-actin	NM_205518	F:ACGTCTCACTGGATTTCGAGCAGG R:TGCATCCTGTCAGCAATGCCAG	298

### Statistics

Data are expressed as means ± standard deviation. Statistical significance was performed using a two-tailed Student’s test. *P <* 0.05 were considered statistically significant, ^*^P < 0.05, ^**^P < 0.01 vs. control 1; ^#^*P <* 0.05, ^##^*P <* 0.01 vs. control 2. SPSS 24.0 software (SPSS, Chicago, USA) was used for statistical analysis. The measured data were statistically ploted with GraphPad Prism 8.0 software.

## Supplementary Information

**Additional file 1.**

## Data Availability

All data generated or analyzed during this study are included in this published article.

## Abbreviations

HS: Heat stress
HSP70: Heat shock protein 70
WG: weight gain
FCR: Feed conversion rate
PRRs: Pattern recognition receptors
TLR4: Toll-like receptor 4
NF-κB: Nucleus factor kappa B
NLRP3: Nucleotide-binding oligomerization domain-like Receptor Family, Pyrin Domain Containing 3
