# Supplementary material for: Chronic heat stress promotes liver inflammation in broilers via enhancing NF-κB and NLRP3 signaling pathway
Source: BMC Vet Res. 2022 Jul 23;18:289. doi: 10.1186/s12917-022-03388-0 (PMC9308265; doi:10.1186/s12917-022-03388-0)
Supplement: Supplementary file 1 — Additional file 1. [file 12917_2022_3388_MOESM1_ESM.docx]

**The images of the original blots.**

Response: The blots had been cut prior to hybridisation with antibodies. The images we provided were the original images of full-length blots in each protein with marker in the left of the blots.

1. Figure3A:IL-6


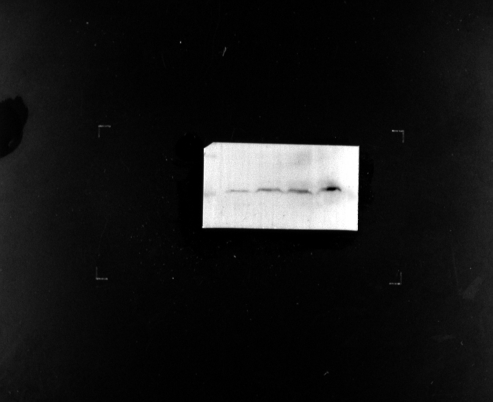


1. Figure3A:TNF-α（left band）


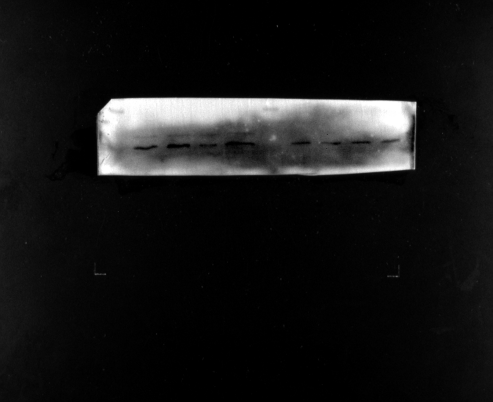


1. Figure3A: GAPDH (left band)





1. Figure4A: NLRP3 (left band)


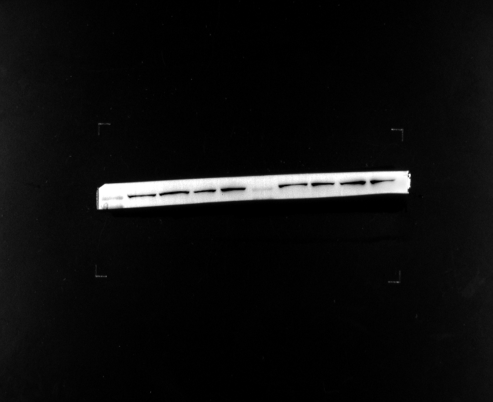


1. Figure4A: Caspase-1 (left band)


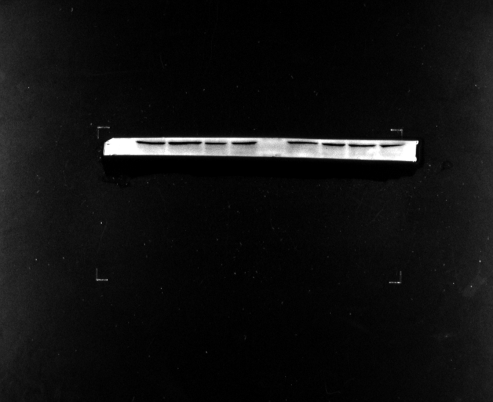


1. Figure4A: Pro-IL-1β (left band)


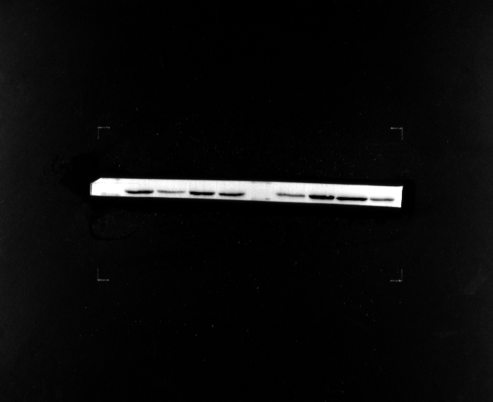


1. Figure4A: Cleaved-IL-1β (left band)


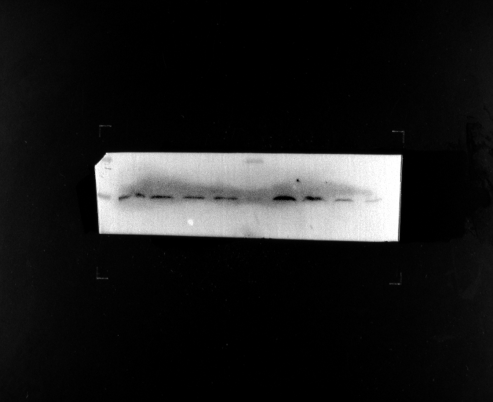


1. Figure4A: GAPDH (right band)





1. Figure5A: P-NF-κB P65 (right band)


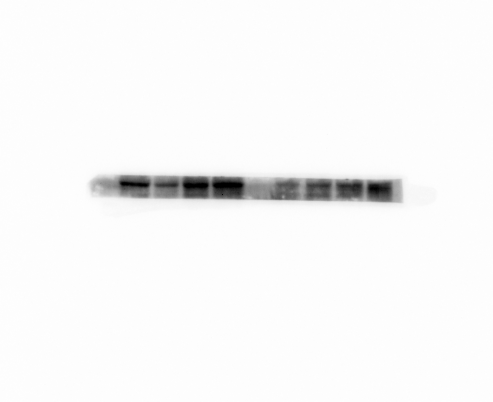


1. Figure5A: NF-κB P65 (left band)


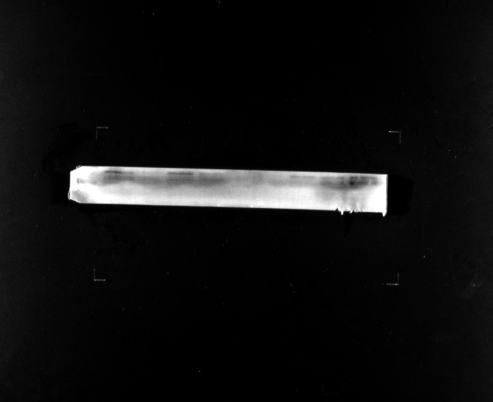


1. Figure5A: P- IκB-α (right band)


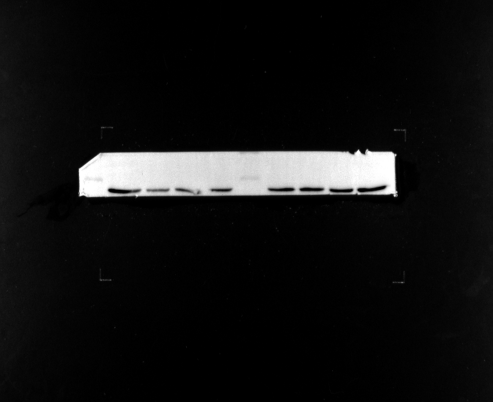


1. Figure5A: IκB-α (left band)


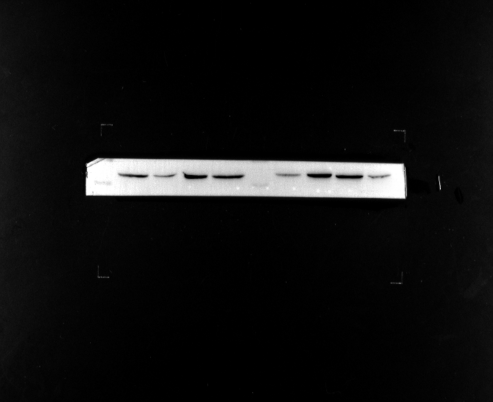


1. Figure5A: GAPDH(right band)
